# Editorial: Challenges, opportunities & outcomes of patient-oriented research in learning health systems

**DOI:** 10.3389/frhs.2026.1789698

**Published:** 2026-02-25

**Authors:** Kiran Pohar Manhas, Karen Benzies, Maria Santana, Tracy Wasylak

**Affiliations:** 1 Partnerships & Innovation, Acute Care Alberta, Calgary, AB, Canada; 2 Faculty of Nursing, University of Alberta, Edmonton, AB, Canada; 3 Faculty of Nursing, University of Calgary, Calgary, AB, Canada; 4 Department of Community Health Sciences and department of Paediatrics, Cumming School of Medicine, University of Calgary, Calgary, AB, Canada; 5 Health Canada, Ottawa, ON, Canada

**Keywords:** learning health system, methodology, organization, patient engagement (PE), patient-Oriented research

## Introduction

This Research Topic brings together scholarly manuscripts that illuminate the evolving intersection of patient-oriented research (POR) and learning health systems (LHS). LHSs mobilize data, incentives, scientific evidence, and organizational culture toward continuous learning, improvement, and innovation. POR meaningfully engages patients, families, communities, and the public throughout the research process to generate knowledge that is relevant, actionable, and responsive to real-world needs. When embedded within LHSs, POR offers a powerful mechanism for harmonizing patient-centred, evidence-informed decision-making across health systems.

Collectively, these articles examine the challenges, opportunities, and outcomes of integrating POR within LHSs. They highlight: (a) innovative participatory methods that advance learning and improvement; (b) infrastructures and governance mechanisms that support sustainable engagement; and (c) examples of POR that identify priorities of marginalized and equity-deserving populations.

## Novel methods in POR & LHSs

Several contributions demonstrate how innovative participatory approaches can advance learning and transformation within LHSs. Aghabayli et al., describe a study co-designed, implemented, and disseminated by individuals with lived experience using the Patient and Community Engagement Research (PaCER) SET–COLLECT–REFLECT process. Examining healthcare navigation programs, the study centres perspectives of both navigators and those being navigated. Identified challenges including limited training, inconsistent funding, communication gaps, and unmet mental health needs and highlight opportunities for system-level improvements, particularly in addressing complexity and equity-related barriers within Alberta's LHS.

Focusing on sustainability and scale, Shahid et al. present a mission-driven approach to patient-oriented commercialization. Through extensive co-design with patients and families, the authors developed and evaluated a family-integrated care model for neonatal intensive care units (FICare, now Merge™). Recognizing that traditional research funding was insufficient for long-term sustainment, a social enterprise model was adopted to ethically commercialize the intervention. This approach prioritized transparency, reinvestment, and stewardship, illustrating commercialization as a viable, though complex, pathway for sustaining POR informed innovations in LHSs.

Participatory design methods are further explored by Lin et al., who describe the use of storytelling and a Design Jam within a large health authority in British Columbia to co-develop a vision for a community-centred LHS. Across two sessions involving multidisciplinary stakeholders, these methods supported idea generation, convergence, and collective sense-making, resulting in actionable strategies to strengthen community involvement. The authors offer practical recommendations to enhance participation, foster equity, and embed community responsiveness in LHS transformation efforts.

## LHS infrastructure to advance POR

Beyond methods, several articles emphasize the role of infrastructure and governance in sustaining POR within LHSs. Giacomantonio et al. present a narrative review of engagement activities across existing and emerging LHSs, identifying 192 distinct activities involving patients, caregivers, communities, and the public. While the review highlights a lack of detailed reporting on engagement practices, it demonstrates considerable diversity in theoretical perspectives, timing, and contributor roles, underscoring multiple pathways for institutionalizing POR.

At a national level, Alhuseini et al. illustrate how a government-mandated patient experience survey has been leveraged for system monitoring and improvement in Saudi Arabia. Capturing patient experiences across multiple care domains, the survey has informed targeted initiatives to enhance professional skills through training and education, demonstrating how patient feedback can function as a core learning mechanism within an LHS.

Mork et al. examine a provincial, integrated LHS that has embedded patient engagement and POR across planning, co-design, implementation, and decision-making. Key enablers include provincial networks, executive sponsorship, and an integrated electronic medical record. Persistent challenges remain, particularly in advancing equity, diversity, and inclusion and sustaining engagement during periods of significant organizational change.

Complementing these system-level perspectives, Wilson et al. reflect on the co-development of a validated parent experience measure in neonatal intensive care units. Their findings highlight the importance of governance, reflexivity, adaptive workflows, and constructive approaches to dissent. Reciprocity and long-term partnership emerge as central to sustaining POR within LHSs.

## POR & LHS for marginalized communities

Engaging marginalized, equity-deserving, and underserved populations remains a critical challenge for POR and LHSs. Several studies in this Topic explicitly centre these groups, demonstrating how inclusive approaches can enhance relevance, equity, and system learning.

Naqvi et al. use a James Lind Alliance approach to prioritize research uncertainties related to mental health among immigrant populations. By engaging immigrant youth as research partners, the study identifies culturally specific experiences and structural barriers, including language, stigma, and limited awareness of resources. These findings illustrate how POR can inform more responsive and adaptive LHSs.

Addressing another underrepresented population, Nagra et al. focus on injured workers experiencing delays in return to work (RTW). The authors argue that POR approaches can better align RTW programs with workers lived experiences, improve coordination across service providers, and enhance the effectiveness and durability of RTW initiatives.

Hecker et al. examine person-centred and integrated care among individuals living with chronic kidney disease using the Rainbow Model of Integrated Care Measurement tool. Comparing perspectives of patients, providers, and caregivers, the study underscores the importance of including caregivers—often underrepresented in POR—to strengthen integrated care and outcomes.

Finally, Kemp et al. explore the experiences of adults who leave hospital against medical advice (LAMA) through co-designed patient-centred quality indicators. LAMA patients report consistently poorer experiences across multiple dimensions of care and are more likely to belong to marginalized groups. The findings highlight the need for respectful, flexible, and empathetic strategies to reduce the frequency and adverse outcomes of LAMA discharges.

## Summary

Taken together, the contributions to this Research Topic demonstrate that embedding POR within LHSs produces meaningful outcomes for system learning, performance, and equity, while also revealing ongoing implementation challenges. Across diverse contexts, POR enhances the relevance and usability of evidence, strengthens feedback loops, and supports more adaptive and legitimate decision-making.

Critically, the studies show that impact depends on moving beyond episodic engagement toward sustained partnerships supported by governance, resources, and accountability. Innovative methods, enabling infrastructures, and inclusive approaches, particularly those centring marginalized and equity-deserving populations, emerge as key levers for translating POR into improved health system outcomes. Collectively, these contributions advance the evidence base for building LHSs that learn with, and from, the people they serve, aligning closely with the mission to promote impactful, open, and patient-centred health research.

